# First-principles investigation of the microscopic mechanism of the physical and chemical mixed adsorption of graphene on metal surfaces

**DOI:** 10.1039/c9ra07111c

**Published:** 2019-10-14

**Authors:** Xin Zhang, Shaoqing Wang

**Affiliations:** Shenyang National Laboratory for Materials Science, Institute of Metal Research, Chinese Academy of Sciences 110016 Shenyang Liaoning China xzhang17b@imr.ac.cn; School of Materials Science and Engineering, University of Science and Technology of China 110016 Shenyang Liaoning China

## Abstract

The binding energy, bond length, projected density of states and differential charge density of graphene–metal interfaces are investigated using a first-principles method in which a single layer graphene is adsorbed on the low-index metal surfaces such as the (111), (110) and (100) surfaces. The bond length results show the graphene sheet has a different degree of buckling after graphene is adsorbed on the (110) and (100) surfaces of metals. The projected density of states and the differential charge density results confirm the adsorption of graphene on the Ni(111), Co(111), Ni(110), Co(110) and Cu(110) surfaces is chemisorption due to the strong orbital coupling effect and the obvious charge accumulation between the carbon and metal atoms, while the adsorption of graphene on the Cu(111) surface is physical adsorption owing to the absence of the orbital coupling effect and the charge accumulation between the carbon and Cu atoms. Interestingly, the adsorption of graphene on the (100) surface of Ni, Co and Cu is all physical and chemical mixed adsorption because there are the strong orbital coupling effect and the apparent charge accumulation between the carbon and metal atoms in some parts of these surfaces while there are almost no orbital coupling effects and charge accumulation between the carbon and metal atoms in other parts.

## Abstract 2

Unlike (111) and (110) surfaces, we find graphene is both physically and chemically adsorbed on the (100) surface of metals.

## Graphical abstract

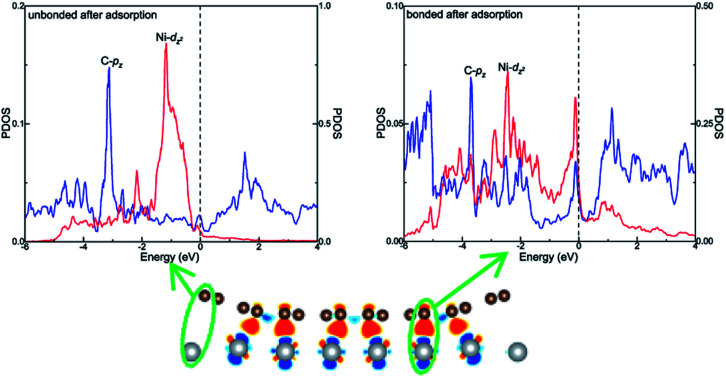

## Introduction

Graphene, a two-dimensional single atomic layer structure which is composed of sp^2^ carbon atoms arranged in a honeycomb crystal lattice, has attracted tremendous attention and research interest since it was prepared successfully by mechanical exfoliation from graphite in 2004.^1,2^ Graphene essentially displays many intriguing and peculiar properties such as a theoretically large surface area (∼2630 m^2^ g^−1^),^3^ high room temperature charge carrier mobility (∼1 × 10^5^ cm^2^ V^−1^ s^−1^),^4^ excellent mechanical properties^5^ including a Young's modulus of 1.0 TPa and fracture strength of 125 GPa, high thermal conductivity (∼2000 to 5000 W m^−1^ K^−1^),^6,7^ capacity to sustain large electrical current density (10^8^ A cm^−2^)^8^ and so on. These unique properties of graphene make it a hot research topic in the field of materials science, and endow graphene-based composites with better properties.^9^ Graphene–metal composites are an important part of the research on graphene-based composites, mainly including graphene-loaded metal nanoparticle composites and graphene–reinforced metal matrix composites. Graphene-loaded metal nanoparticle composites utilize graphene as the carrier to load metal nanoparticles in order to enhance the activity and dispersion of nanoparticles, which are applied in a broad range of fields such as catalysis,^10–12^ sensors,^13,14^ spectroscopy^15,16^ and so on. Graphene–reinforced metal matrix composites incorporate graphene sheets into the metal matrix, which can greatly enhance mechanical properties of metal materials without affecting or even improving the thermal and electrical properties of materials.^17–19^ Obviously, the interactions between graphene and metals will play a crucial role in the properties of graphene-based composites. Therefore, it is essential to study the interactions between graphene and different metal surfaces. There have been numerous experimental and theoretical studies on graphene contacted to metals such as Ni, Co, Ru, Rh, Pd, Ir, Pt and Cu.^20–37^ Experimental studies about the growth of graphene on metal close-packed surface such as Ni(111), Cu(111) and Ru(0001) rank first.^38–41^ Secondly, experimental studies about the growth of graphene on other low-index metal surfaces such as Ni(100), Rh(100) and Pt(110) have also been reported.^42–44^ However, theoretical studies on the interfaces between graphene and metals mainly focus on the metal close-packed surface such as Ni(111), Co(111), Cu(111) and so on.^28–37^ In 2018, Mafra *et al.* not only investigated different growth mechanisms of graphene on Ni(100), (110) and (111) surface by optical microscopy, Raman spectroscopy and optical transmission but also provided an atomistic model of the processes involved to support the experimental results by density functional theory calculations.^43^ Theoretical studies on the adsorption of graphene on the (110) and (100) surfaces of other metals except Ni are rarely reported.

The (111), (110) and (100) surfaces are the most basic and important low-index metal surfaces. In particular, the close-packed structure of the (111) surface of Ni, Co and Cu has been commonly used to make graphene–metal contacts owing to their structural resemblance. The difference is the adsorption of graphene on the (111) surface of Ni and Co is chemisorption while the adsorption of graphene on the Cu(111) surface is physical adsorption. However, the lack of hexagonal symmetry of (110) and (100) surfaces results in less theoretical studies on graphene–metal contacts. Therefore, in this work, in order to explore the adsorption mechanism of graphene on metal surfaces, taking Ni, Co and Cu as examples, the interfaces between graphene and low-index metal surfaces such as (111), (110) and (100) surfaces are investigated by using first-principles calculations at the level of density functional theory (DFT). The results obtained by the present study are not only expected to explain the adsorption mechanism of graphene on the metal surfaces, but also to provide help for the research of graphene-based composites and carbon nanomaterials.

## DFT calculations

**Fig. 1:**
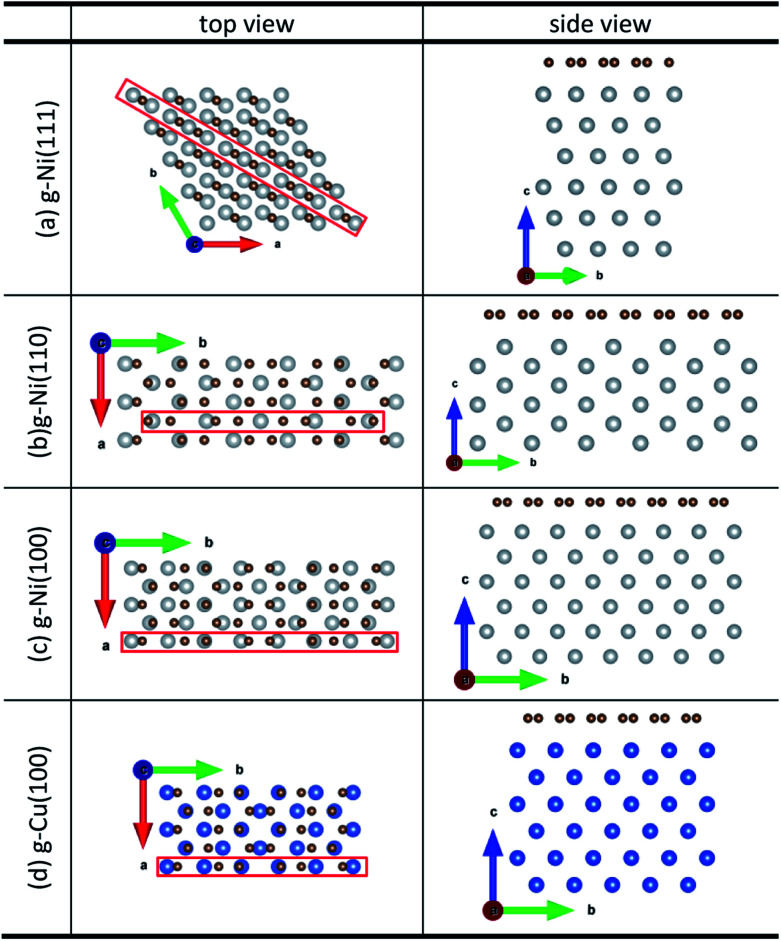
The top and side views of initial configurations ((a) graphene–Ni(111), (b) graphene–Ni(110), (c) graphene–Ni(100) and (d) graphene–Cu(100)).

Density function theory (DFT) calculations based on plane-wave basis sets of 500 eV cut-off energy were performed with the Vienna *ab initio* simulation package (VASP).^44,45^ The local density approximation (LDA)^46^ was used to describe the exchange correlation effect because of the better performance of LDA compared with the generalized gradient approximation (GGA) in predicting binding behaviour for interfaces between carbon nanostructures and metals.^26,47^ The projector-augmented wave (PAW) was used to describe the electron–ion interactions.^48,49^ Metal surface is constructed in a supercell as a finite number of layers of metal plus a region of vacuum repeated periodically in the direction perpendicular to the layers. The supercell used to model the graphene–metal interface is constructed from a slab consisting of six layers of metal atoms, with a graphene layer adsorbed from the top side of the slab and a vacuum region of 15 Å. Considering that the metal substrate is usually much thicker than the upper layer film and it is usually the film that trends to match the lattice constant (LC) of the metal substrate in experiment,^50,51^ the lattice constant of metal unit cells is fixed to construct the interface supercell. Therefore, taking the metal Ni as an example, the 4 × 4 graphene unit cell is adjusted to the 4 × 4 unit cell of Ni(111) surface as shown in Fig. 1(a); the 
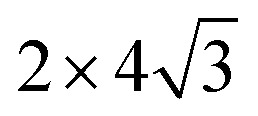
 graphene unit cell is adjusted to the 
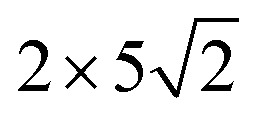
 unit cell of Ni(110) surface as shown in Fig. 1(b); the 
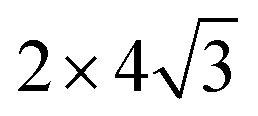
 graphene unit cell is adjusted to the 2 × 7 unit cell of Ni(100) surface as shown in Fig. 1(c); while for graphene–Cu(100) interface, the 
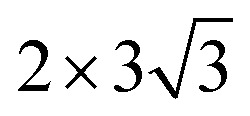
 graphene unit cell is adjusted to the 2 × 5 unit cell of Cu(100) surface in order to build a better matching model as shown in Fig. 1(d). The approximation made by the matching procedure is reasonable since the mismatch with the lattice constant of the graphene sheet is only 0.629–3.825%, as seen in Table 1. A dipole correction is applied to avoid spurious interactions between periodic images of the slab.^52^ The electronic self-convergence criterion is set to 1.0 × 10^−5^ eV and the forces on all the atoms are converged to within 0.01 eV Å^−1^ with respect to ionic relaxation.

**Table 1:** The calculated lattice constant of graphene and metal supercell (*a*_g_, *b*_g_ and *a*_m_, *b*_m_), lattice strain of graphene (*a*% and *b*%), calculated average equilibrium interfacial distance (*d*_eq_), binding energy (*E*_b_) and the maximum buckling on the graphene layer are listed

	g–Ni(111)	g–Co(111)	g–Cu(111)	g–Ni(110)	g–Co(110)	g–Cu(110)	g–Ni(100)	g–Co(100)	g–Cu(100)
*a*_g_ (Å)	9.784	9.784	9.784	4.892	4.892	4.892	4.892	4.892	4.892
*b*_g_ (Å)	9.784	9.784	9.784	16.947	16.947	16.947	16.947	16.947	12.710
*a*_m_ (Å)	9.666	9.527	9.968	4.833	4.763	4.984	4.833	4.763	4.984
*b*_m_ (Å)	9.666	9.527	9.968	17.086	16.841	17.621	16.915	16.672	12.460
*a*%	1.221	2.698	−1.846	1.221	2.698	−1.846	1.221	2.698	−1.846
*b*%	1.221	2.698	−1.846	−0.814	0.629	−3.825	0.189	1.649	2.006
*d*_eq_ (Å)	2.016	1.976	3.004	2.004	2.028	2.257	2.165	2.209	2.734
*d* ^Ref^_eq_ (Å)	2.05^a^	2.05^a^	3.26^a^	2.03^b^	—	—	2.13^b^	—	—
*E*_b_ (eV per C)	0.135	0.259	0.022	0.178	0.221	0.017	0.175	0.162	0.028
*E* ^Ref^_b_ (eV per C)	0.125^a^	0.160^a^	0.033^a^	0.209^b^	—	—	0.180^b^	—	—
Buckling (Å)	0.0010	0.0102	0.0006	0.2016	0.3694	0.1192	1.0178	1.2743	0.8924
Buckling^Ref^ (Å)	0.00^a^	—	—	0.29^b^	—	—	0.69^b^	—	—

a Ref. 28.

b Ref. 43.

## Results and discussions

### Graphene–metal binding

The average equilibrium interfacial distance (*d*_eq_), the binding energy (*E*_b_) for graphene adsorbed on the (111), (110) and (100) surfaces of metals and the maximum buckling on the graphene layer studied in this paper are listed in Table 1. It can be seen our calculated results are slightly different from those reported in the literatures,^28,43^ but the overall trend is basically the same. We believe the difference in results may be due to the following reasons. Firstly, the supercells we constructed are larger than those in the literature^28,53^ when graphene is adsorbed on the (111) surface of metals. Besides, the adsorption configuration of graphene on the (111) surface of metals is different from that in the literature,^28^ but this configuration has been confirmed by experiments.^34^ The binding energy in the paper was calculated by underlying formula*E*_b_ = (*E*_graphene_ + *E*_metal surface_) − *E*_graphene–metal interface_where, *E*_graphene_, *E*_metal surface_ and *E*_graphene–metal interface_ represent the total energy of the bare slab, the isolated graphene, and the adsorption system, respectively. According to the definition, a positive *E*_b_ indicates the adsorption system should be stable. Buckling is defined as the coordinate difference of carbon atoms along the *z* direction.

In our calculations, all graphene-metal interface models can be confirmed to be stable according to the definition of the binding energy. All the initial configurations are shown in Fig. 1. When graphene is adsorbed on the (111) surface of metals, the average equilibrium interfacial distance (*d*_eq_) and the binding energy (*E*_b_) show the adsorption types of graphene on the (111) surface of metals can be divided into chemisorption and physical adsorption according to the literature.^27^ To be more specific, when *d*_eq_ is less than 2.3 Å and *E*_b_ is greater than 0.1 eV per C, the adsorption of graphene on the (111) surface of metals is chemisorption, while *d*_eq_ is greater than 3 Å and *E*_b_ is less than 0.04 eV per C, the adsorption of graphene on the (111) surface of metals is physical adsorption. Combining the above criterion of adsorption type and our calculation results, it can be found the adsorption of graphene on the (111) surface of Ni and Co is chemisorption, while the adsorption of graphene on Cu(111) surface is physical adsorption. However, for graphene adsorbed on the (110) surface of metals, the average equilibrium interfacial distances are less than 2.3 Å, the binding energies of the graphene–Ni(110) interface and the graphene–Co(110) interface are greater than 0.1 eV per C while the binding energy of the graphene–Cu(110) interface is less than 0.04 eV per C. Unlike graphene adsorbed on the (111) surface of metals, the graphene sheet shows a certain degree of buckling. Therefore, the adsorption type of graphene on the (110) surface of metals needs to be further investigated. In terms of graphene adsorbed on the (100) surface of Ni and Co, the average equilibrium interfacial distances are less than 2.3 Å and the binding energies are 0.175 and 0.162 eV per C, respectively. Notably, when graphene is adsorbed on Cu(100) surface, the average equilibrium interfacial distance is more than 2.3 Å but less than 3 Å, and the binding energy is still less than 0.04 eV per C. However, the adsorption type of graphene on the (100) surface of metals also needs to be further investigated because the graphene sheet has a greater degree of buckling after graphene is adsorbed on the (100) surface. In conclusion, it is unreasonable to analyse the adsorption types of graphene on the (110) and (100) surfaces of metals by combining the binding energy and the average equilibrium interfacial distance due to no consideration for the buckling on the graphene sheet. In order to determine the adsorption types of graphene on different surfaces of Ni, Co and Cu more accurately, the bond lengths (*r*) between carbon and metal atoms at the interface can be used as a new criterion since the adsorption types of graphene on the (111) surface of Ni, Co and Cu can be determined. And the bond length (*r*) analysis will be discussed in the following paragraphs.

### Bond length analysis

**Fig. 2:**
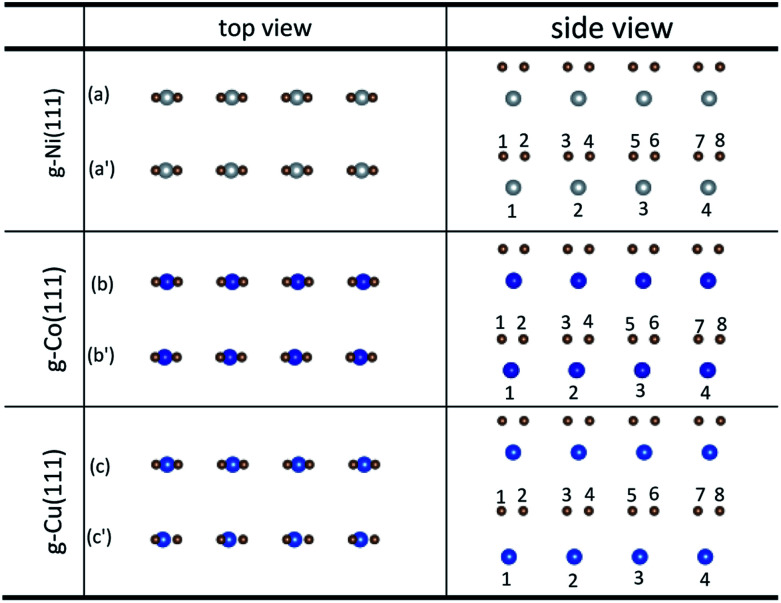
The top and side views of local configurations before ((a), (b) and (c)) and after ((a′), (b′) and (c′)) the adsorption of graphene on the (111) surface of Ni, Co and Cu.

The initial configurations were constructed by matching graphene to metal surfaces as shown in Fig. 1. The relative positions and the bond lengths (*r*) of carbon and metal atoms have changed to achieve the lowest energy state after graphene is adsorbed on metal surfaces. Local configurations are selected as the research object in order to better observe and compare the relative positions and the bond lengths (*r*) between carbon and metal atoms. It should be noted that local configurations are taken from the red line frame areas in Fig. 1. The top and side views of local configurations induced by the adsorption of graphene on the (111) surface of metals are shown in Fig. 2. The top and side views show the relative positions of carbon and metal atoms have changed slightly after optimization. The bond lengths (*r*) between carbon and metal atoms induced by the adsorption of graphene on the (111) surface of Ni, Co and Cu are shown in Table 2. Therefore, the new criterion for adsorption types of graphene on Ni, Co and Cu surfaces is as follows. When the bond lengths (*r*) between carbon and metal atoms at the interface are about 2.2 Å, indicating carbon atoms can form covalent bonds with metal atoms, that's to say, the adsorption of graphene on Ni, Co and Cu surfaces is chemisorption, while the bond lengths (*r*) between carbon and metal atoms are larger than 3 Å, indicating carbon atoms don't form covalent bonds with metal atoms, that's to say, the adsorption of graphene on Ni, Co and Cu surfaces is physical adsorption.

**Table 2:** The bond lengths of carbon and metal atoms induced by the adsorption of graphene on the (111) surface of Ni, Co and Cu

*r* (Å)	C–Ni(111)	C–Co(111)	C–Cu(111)
*r*_1–1_	2.116	2.068	3.043
*r*_2–1_	2.149	2.117	3.152
*r*_3–2_	2.116	2.068	3.043
*r*_4–2_	2.149	2.117	3.152
*r*_5–3_	2.116	2.068	3.043
*r*_6–3_	2.149	2.117	3.152
*r*_7–4_	2.116	2.068	3.043
*r*_8–4_	2.149	2.117	3.152

**Fig. 3:**
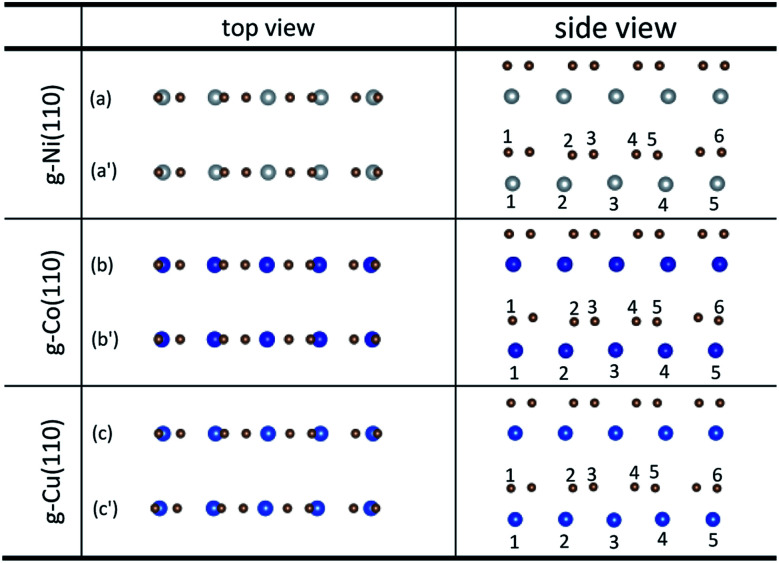
The top and side views of local configurations before ((a), (b) and (c)) and after ((a′), (b′) and (c′)) the adsorption of graphene on the (110) surface of Ni, Co and Cu.

Unlike graphene adsorbed on the (111) surface of Ni, Co and Cu, the graphene sheet shows a certain of buckling after graphene is adsorbed on the (110) surface of metals from the side views as shown in Fig. 3. Table 3 gives the bond lengths (*r*) of carbon and metal atoms induced by the adsorption of graphene on the (110) surface of metals. The maximum and minimum bond lengths (*r*) between carbon and Ni atoms are 2.357 Å and 2.009 Å, respectively. And the maximum and minimum bond lengths (*r*) between carbon and Co atoms are 2.398 Å and 2.039 Å, respectively. According to the new criterion mentioned above, it is known that the adsorption of graphene on the (110) surface of Ni and Co is chemisorption. However, for the graphene–Cu(110) interface, the minimum bond length (*r*) between carbon and Cu atoms is 2.218 Å, which is very close to 2.2 Å, while the maximum bond length (*r*) between carbon and Cu atoms is 2.770 Å, which is slightly larger than 2.2 Å, but less than 3 Å. In general, the adsorption of graphene on Cu(110) surface is also chemisorption, but the interaction between carbon and Cu atoms is slightly weaker than that between carbon and Ni (or Co) atoms.

**Table 3:** The bond lengths of carbon and metal atoms induced by the adsorption of graphene on the (110) surface of Ni, Co and Cu

*r* (Å)	C–Ni(110)	C–Co(110)	C–Cu(110)
*r*_1–1_	2.078	2.039	2.218
*r*_2–2_	2.009	2.040	2.270
*r*_3–3_	2.357	2.398	2.770
*r*_4–3_	2.357	2.398	2.770
*r*_5–4_	2.009	2.040	2.270
*r*_6–5_	2.078	2.039	2.218

**Fig. 4:**
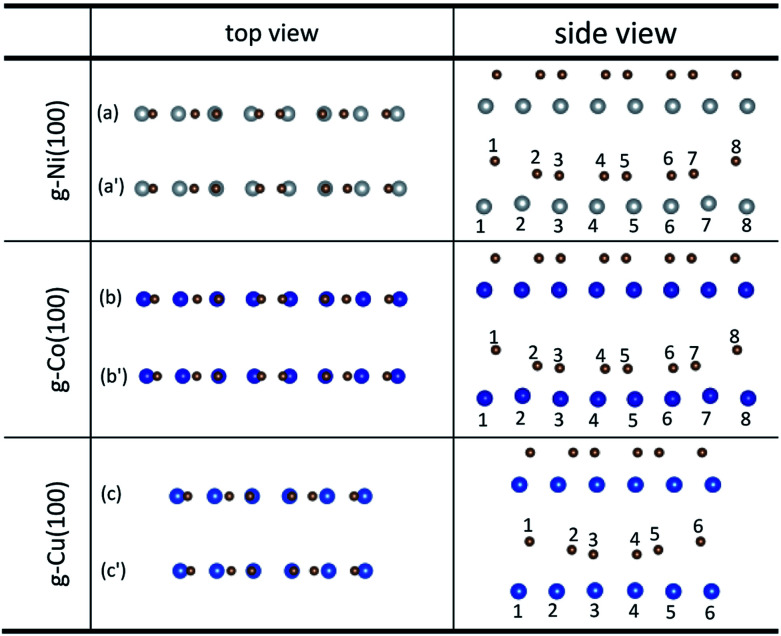
The top and side views of local configurations before ((a), (b) and (c)) and after ((a′), (b′) and (c′)) the adsorption of graphene on the (100) surface of Ni, Co and Cu.

An interesting phenomenon is that the graphene sheet shows a large of buckling after graphene is adsorbed on the (100) surface of Ni, Co and Cu from the side views as shown in Fig. 4. In addition, there are obvious changes in the relative positions of carbon and Ni atoms observed from the top views as shown in Fig. 4. Table 4 shows significant changes in the bond lengths (*r*) of carbon and metal atoms after optimization. The bond lengths (*r*) of majority carbon and Ni atoms are less than 2.2 Å, and the bond lengths (*r*) of minority carbon and Ni atoms are larger than 3 Å. Similarly, the bond lengths (*r*) of majority carbon and Co atoms are about 2.2 Å, and the bond lengths (*r*) of minority carbon and Co atoms are larger than 3 Å. According to the new criterion mentioned above, it can be concluded that the adsorption of graphene on the (100) surface of Ni and Co is physical and chemical mixed adsorption. Unlike the graphene–Ni(100) interface and the graphene–Co(100) interface, the bond lengths (*r*) of minority carbon and Cu atoms are about 2.2 Å, and the bond lengths of some carbon and Cu atoms are close to 3 Å while the bond lengths (*r*) of other carbon and Cu atoms are larger than 3 Å, which indicates the interactions between carbon and Cu atoms are slightly weaker than those between carbon and Ni (or Co) atoms. That's to say, the adsorption of graphene on the Cu(100) surface is the coexistence of physical adsorption and weak chemisorption. In other words, the adsorption of graphene on the (100) surface of Ni, Co and Cu is all physical and chemical mixed adsorption.

**Table 4:** The bond lengths of carbon and metal atoms induced by the adsorption of graphene on the (100) surface of Ni, Co and Cu

*r* (Å)	C–Ni(100)	C–Co(100)	C–Cu(100)
*r*_1–1_	3.001	3.164	3.278
*r*_2–2_	2.130	2.129	2.843
*r*_3–3_	1.970	1.966	2.321
*r*_4–4_	1.998	1.997	2.321
*r*_5–5_	1.998	1.997	2.843
*r*_6–6_	1.970	1.966	3.278
*r*_7–7_	2.130	2.129	—
*r*_8–8_	3.001	3.164	—

According to the geometric analysis above, the adsorption types of graphene on the metal surfaces are initially determined. Chemisorption process will not only have structural changes, such as the bond lengths (*r*) and the relative positions between the carbon and metal atoms, but also have chemical changes, such as the coupling effect between atomic orbitals and charge accumulation between atoms. Therefore, in order to further verify adsorption types of graphene on the metal surfaces, we calculated the projected density of states (PDOS) and the differential charge density in the following paragraphs, respectively.

### Projected density of states (PDOS)

**Fig. 5:**
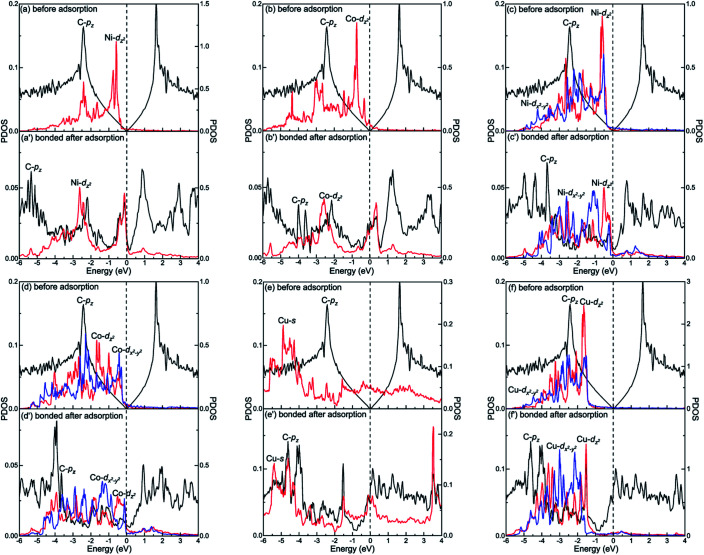
The PDOS of carbon and metal atoms before and after graphene is chemisorbed on the (a and a′) Ni(111), (b and b′) Co(111), (c and c′) Ni(110), (d and d′) Co(110) and (e, e′, f and f′) Cu(110) surfaces. Left-*Y* represents PDOS of carbon atoms and Right-*Y* represents PDOS of metal atoms.

**Fig. 6:**
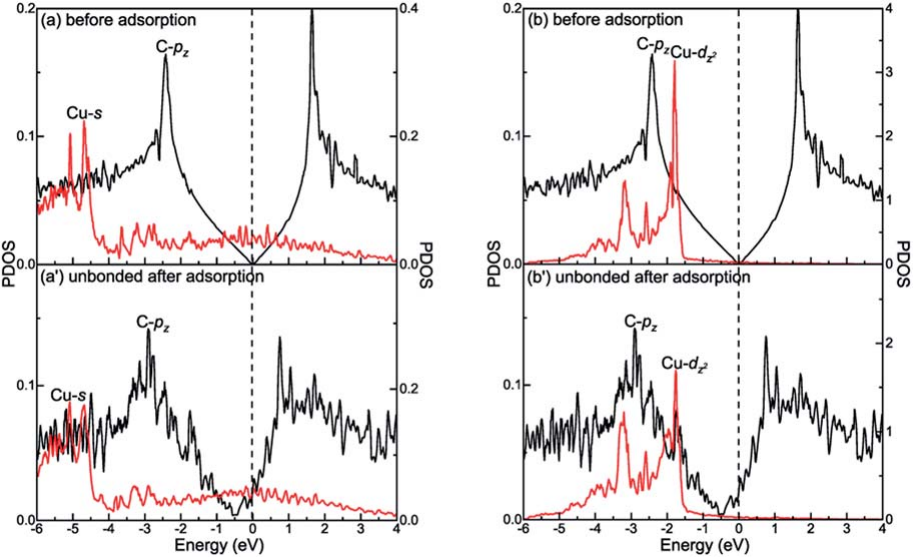
The PDOS of carbon and metal atoms before and after graphene is physically adsorbed on the (a and b) and (a′ and b′) Cu(111) surface. Left-*Y* represents PDOS of carbon atoms and Right-*Y* represents PDOS of metal atoms.

**Fig. 7:**
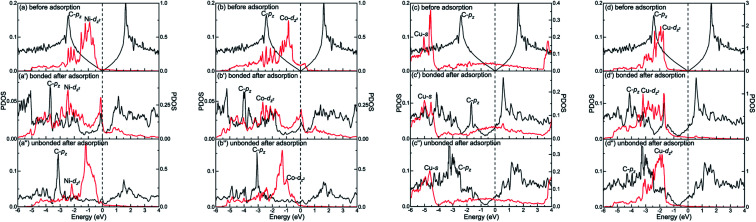
The PDOS of carbon and metal atoms before and after graphene is physically and chemically mixed adsorbed on the (a, a′ and a′′) Ni(100), (b, b′ and b′′) Co(100), and (c–c′′ and d–d′′) Cu(100) surfaces. Left-*Y* represents PDOS of carbon atoms and Right-*Y* represents PDOS of metal atoms.

The different interactions between graphene and the metal surfaces mainly arise from the different properties of metals. Ni atom (3d^8^4s^2^) and Co atom (3d^7^4s^2^) have open d-shell with two and three unpaired electrons, respectively, while Cu atom (3d^10^4s^1^) has fully filled d-orbital and half-filled s-orbital, which means Cu atom has only one unpaired electron. Therefore, the interactions between graphene and Ni (or Co) are stronger. Further investigation of the binding mechanism for graphene–metal interfaces was carried out by analysing the projected density of states (PDOS). Notably, as is known to all, the electronic states of graphene become obviously spin-polarized after it is adsorbed on the different surfaces of Ni and Co. Here, the present study only gives the PDOS of spin-up states in order to simplify the analysis. Fig. 5–7 show the projected densities of states of carbon and metal atoms at the interface before and after graphene is adsorbed on different metal surfaces, respectively. According to the degree of orbital coupling effect, the adsorption types of graphene on Ni, Co and Cu surfaces can be divided into chemisorption, physical adsorption and physical and chemical mixed adsorption.

There exists the strong orbital coupling effect between the carbon and metal atoms after graphene is adsorbed on the Ni(111), Co(111), Ni(110), Co(110) and Cu(110) surfaces, that is to say, the adsorption of graphene on these metal surfaces is chemisorption. In more detail, for the graphene–Ni(111) interface, many overlapping peaks between the C-p_*z*_ orbital and the Ni-d_*z*^2^_ orbital are found in the energy range from −6 to −4.5 eV, −4 to −2 eV and −1 to 0 eV after graphene is adsorbed on the Ni(111) surface as shown in Fig. 5(a′), which shows the formation of covalent bonds between the C-p_*z*_ orbital and the Ni-d_*z*^2^_ orbital. Still, the electron occupied states of the Ni-d_*z*^2^_ orbital increase significantly above the Fermi energy. Similarly, in the case of the graphene–Co(111) interface, the same conclusions can be drawn according to the projected density of states results in Fig. 5(b′). After graphene is adsorbed on the Ni(110) surface, for carbon and Ni atoms near the configuration edge, many overlapping peaks in the energy range from −5 to −3.5 eV, −3 to −1 eV, and −0.5 to 0 eV are found between the C-p_*z*_ orbital and the Ni-d_*z*^2^_ orbital as shown in Fig. 5(c′), indicating the C-p_*z*_ orbital and the Ni-d_*z*^2^_ orbital form covalent bonds. Above the Fermi energy, the electron occupied states of the Ni-d_*z*^2^_ orbital increase obviously. In addition, for carbon and Ni atoms near the configuration centre, a few overlapping peaks between the C-p_*z*_ orbital and the Ni-d_*x*^2^−*y*^2^_ orbital in the energy range from −3 to 0 eV, which indicates the formation of covalent bonds between the C-p_*z*_ orbital and the Ni-d_*x*^2^−*y*^2^_ orbital. Above the Fermi energy, the electron occupied states of the Ni-d_*x*^2^−*y*^2^_ orbital also increase apparently. Similar to the graphene–Ni(110) interface, the same conclusions can also be drawn for the graphene–Co(110) interface according to the projected density of states results in Fig. 5(d′). However, when it comes to the graphene–Cu(110) interface, for carbon and Cu atoms near the configuration edge, a few overlapping peaks are found between the C-p_*z*_ orbital and the Cu-s orbital in the energy range from −5 to −4.5 eV, −3 to −2.5 eV, −2 to −1.5 eV and −0.5 to 0 eV as shown in Fig. 5(e′), showing the formation of covalent bonds between the C-p_*z*_ orbital and the Cu-s orbital. Above the Fermi energy, the electron occupied states of the Cu-s orbital increase. Moreover, some overlapping peaks between the C-p_*z*_ orbital and the Cu-d_*z*^2^_ orbital in the energy range from −5.5 to −3.5 eV and −3 to −1.5 eV as shown in Fig. 5(f′) indicate the C-p_*z*_ orbital and the Cu-d_*z*^2^_ orbital form covalent bonds but the amount of overlapping peaks is less than that between the C-p_*z*_ orbital and the Ni-d_*z*^2^_ (or Co-d_*z*^2^_) orbital, illustrating the interaction between the C-p_*z*_ orbital and the Cu-d_*z*^2^_ orbital is weaker. Above the Fermi energy, the electron occupied states of the Cu-d_*z*^2^_ orbital also increase remarkably. For carbon and Cu atoms near the configuration centre, a few overlapping peaks are found between the C-p_*z*_ orbital and the Cu-d_*x*^2^−*y*^2^_ orbital as shown in Fig. 5(f′) in the energy range from −4.5 to −1.5 eV, demonstrating the C-p_*z*_ orbital and the Cu-d_*x*^2^−*y*^2^_ orbital form covalent bonds. What's more, the electron occupied states of the Cu-d_*x*^2^−*y*^2^_ orbital also increase obviously above the Fermi energy.

However, there is no orbital coupling effect between the carbon and Cu atoms after graphene is adsorbed on the Cu(111) surface. Thus, the adsorption of graphene on the Cu(111) surface is only physical adsorption. More specifically, there are slight changes in the PDOS of the C-p_*z*_ orbital but the projected densities of states of the Cu-s and Cu-d_*z*^2^_ orbitals remain almost unchanged and there are no overlapping peaks found between the C-p_*z*_ orbital and the Cu-s and Cu-d_*z*^2^_ orbitals as shown in Fig. 6(a′) and (b′), which indicates the C-p_*z*_ orbital does not form covalent bonds with the Cu-s and Cu-d_*z*^2^_ orbitals. Still, the electron occupied states of the Cu-s and Cu-d_*z*^2^_ orbitals remain almost unchanged.

Interestingly, there exists the strong orbital coupling effect between the carbon and metal atoms in some parts of the model while there is no orbital coupling effect between the carbon and metal atoms in other parts of the model after graphene is adsorbed on the (100) surface of Ni, Co and Cu. Therefore, the adsorption of graphene on the (100) surface of Ni, Co and Cu is all physical and chemical mixed adsorption. When it comes to the graphene–Ni(100) interface, for carbon and Ni atoms near the configuration centre, there are lots of overlapping peaks found between the C-p_*z*_ orbital and the Ni-d_*z*^2^_ orbital in the energy range from −5.5 to −1.5 eV, and −0.5 to 0 eV as shown in Fig. 7(a′), demonstrating the C-p_*z*_ orbital and the Ni-d_*z*^2^_ orbital form covalent bonds. Still, the electron occupied states of the Ni-d_*z*^2^_ orbital above the Fermi energy increase obviously. However, for carbon and Ni atoms near the configuration edge, there are no overlapping peaks found between the C-p_*z*_ orbital and the Ni-d_*z*^2^_ orbital below the Fermi energy as shown in Fig. 7(a′′), which shows no formation of covalent bonds between the C-p_*z*_ orbital and the Ni-d_*z*^2^_ orbital. Besides, the electron occupied states of the Ni-d_*z*^2^_ orbital above the Fermi energy also remain almost unchanged. Similarly, as far as the graphene–Co(100) interface is concerned, the same conclusions can be drawn according to Fig. 7(b′) and (b′′). However, as far as the graphene–Cu(100) interface is concerned, for carbon and Cu atoms near the configuration centre, several overlapping peaks are found between the C-p_*z*_ orbital and the Cu-s orbital in the energy range from −5.5 to −5 eV and −2 to −1.5 eV as shown in Fig. 7(c′), which indicates the C-p_*z*_ orbital and the Cu-s orbital form covalent bonds. Above the Fermi energy, the electron occupied states of the Cu-s orbital increase. What's more, the overlapping peaks between the C-p_*z*_ orbital and the Cu-d_*z*^2^_ orbital mainly concentrate in the energy range from −4 to −1 eV as shown in Fig. 7(d′), which also shows the formation of covalent bonds between the C-p_*z*_ orbital and the Cu-d_*z*^2^_ orbital. Still, the electron occupied states of the Cu-d_*z*^2^_ orbital increase apparently above the Fermi energy. It's worth noting that the amount of overlapping peaks between the C-p_*z*_ orbital and the Cu-d_*z*^2^_ orbital is less than that of the central carbon atoms adsorbed on the Ni(100) and Co(100) surfaces, which indicates the interaction between the C-p_*z*_ orbital and the Cu-d_*z*^2^_ orbital is weaker. However, for carbon and Cu atoms near the configuration edge, there are no overlapping peaks found between the C-p_*z*_ orbital and the Cu-s and Cu-d_*z*^2^_ orbitals as shown in Fig. 7(c′′) and (d′′), showing the C-p_*z*_ orbital does not form covalent bonds with the Cu-s and Cu-d_*z*^2^_ orbitals. Besides, the electron occupied states of the Cu-s and Cu-d_*z*^2^_ orbitals remain almost unchanged.

### Differential charge density

**Fig. 8:**
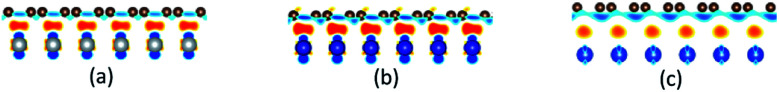
The differential charge density plots induced by the adsorption of graphene on (a) Ni(111), (b) Co(111), (c) Cu(111).

**Fig. 9:**
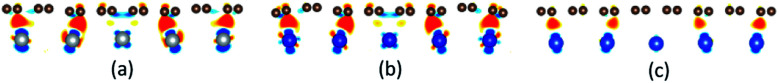
The differential charge density plots induced by the adsorption of graphene on (a) Ni(110), (b) Co(110), (c) Cu(110).

**Fig. 10:**
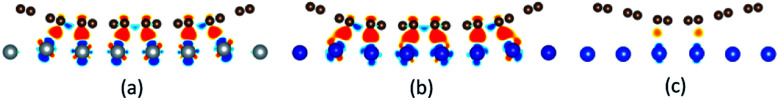
The differential charge density plots induced by the adsorption of graphene on (a) Ni(100), (b) Co(100), (c) Cu(100).

The differential charge density has been calculated to further confirm the adsorption types of graphene on the metal surfaces. The differential charge density plots induced by the adsorption of graphene on (111), (110) and (100) surfaces of metals are shown in Fig. 8–10, respectively. The red/blue colours mark an increase/decrease of the charge density. In addition, iso surfaces correspond to 5 × 10^−3^ e Å^−3^ and the saturation levels are from −0.01 to 0.01 e Å^−3^ for Fig. 8–10 except for Fig. 8(c). Iso surfaces correspond to 5 × 10^−4^ e Å^−3^ and the saturation levels are from −0.001 to 0.001 e Å^−3^ for Fig. 8(c). In the case of the graphene–Ni(111) interface as shown in Fig. 8(a), lots of charge accumulation is found between the carbon and Ni atoms at the interface, which shows the carbon and Ni atoms form covalent bonds. In other words, the adsorption of graphene on the Ni(111) surface is chemisorption adsorption. For the graphene–Co(111) interface as shown in Fig. 8(b), the differential charge density result of the graphene–Co(111) interface is similar to that of the graphene–Ni(111) interface, that's to say, the adsorption of graphene on the Co(111) surface is also chemisorption. By contrast, there is a little charge accumulation between the carbon and Cu atoms at the graphene–Cu(111) interface after graphene is adsorbed on the Cu(111) surface as shown in Fig. 8(c), illustrating there are no covalent bonds between the carbon and Cu atoms. Therefore, the adsorption of graphene on the Cu(111) surface is only physical adsorption.

Notably, there are few reports about the differential charge densities after graphene is adsorbed on the (110) and (100) surfaces of metals. For the graphene–Ni(110) interface as shown in Fig. 9(a), there exists a lot of charge accumulation between the carbon and Ni atoms at the interface, which confirms the carbon and Ni atoms form covalent bonds. That's to say, the adsorption of graphene on the Ni(110) surface is chemisorption. When it comes to the graphene–Co(110) interface as shown in Fig. 9(b), the same conclusion can be drawn that the adsorption of graphene on the Co(110) interface is also chemisorption. In terms of the graphene–Cu(110) interface as shown in Fig. 9(c), there also exists many charge accumulation between the carbon and Cu atoms at the interface. The result shows the carbon and Cu atoms also form covalent bonds, which confirms the adsorption of graphene on the Cu(110) surface is chemisorption but the interaction at the graphene–Cu(110) interface is weaker than that at the graphene–Ni(110) interface and at the graphene–Co(110) interface. Therefore, to be more accurate, the adsorption of graphene on the Cu(110) surface is weak chemisorption.

An interesting phenomenon is that the graphene sheet shows a large of buckling after graphene is adsorbed on the (100) surface of Ni, Co and Cu, which makes the interactions between the carbon and metal atoms have some differences. In terms of the graphene–Ni(100) interface as shown in Fig. 10(a), for carbon and Ni atoms near the configuration edge, there is almost no charge transfer found between them, illustrating the interactions between them are only van der Waals force. Therefore, the adsorption of the carbon atoms near the configuration edge on the Ni(100) surface is physical adsorption. However, for carbon and Ni atoms near the configuration centre, there is a lot of charge accumulation between them at the interface, which explains why the carbon and Ni atoms form covalent bonds. In other words, the adsorption of the carbon atoms near the configuration centre on the Ni(100) surface is chemisorption. Therefore, the adsorption of graphene on the Ni(100) surface is the coexistence of physical adsorption and chemisorption. Similar differential charge density result can be found for the graphene–Co(100) interface as shown in Fig. 10(b), that's to say, the adsorption of graphene on the Co(100) surface is also physical and chemical mixed adsorption. Compared with the graphene–Ni(100) interface and graphene–Co(100) interface, for the carbon and Cu atoms near the configuration edge as shown in Fig. 10(c), almost no charge accumulation is found between them at the interface, which shows the carbon and Cu atoms don't form covalent bonds, that is, the adsorption of the carbon atoms near the configuration edge on the Cu(100) surface is physical adsorption. On the contrary, for carbon and Cu atoms near the configuration centre, charge accumulation is found at the interface, illustrating that the carbon and Cu atoms form covalent bonds. In other words, the adsorption of the carbon atoms near the configuration centre on the Cu(100) surface is chemisorption. Therefore, the adsorption of graphene on the (100) surface of Ni, Co and Cu is all physical and chemical mixed adsorption.

## Conclusions

We presented a first-principles investigation of the binding energy, the bond length, the projected density of states and the differential charge density of the graphene–metal interfaces. The interfaces between graphene and low-index metal surfaces such as (111), (110) and (100) surfaces are mainly discussed in this paper. We find that the graphene sheet has a different degree of buckling after graphene is adsorbed on the (110) and (100) surfaces of Ni, Co and Cu. The projected density of states and the differential charge density results confirm the adsorption of graphene on the Ni(111), Co(111), Ni(110), Co(110) and Cu(110) surfaces is chemisorption due to the strong orbital coupling effect and the obvious charge accumulation between the carbon and metal atoms, while the adsorption of graphene on the Cu(111) surface is physical adsorption owing to the absence of the orbital coupling effect and the charge accumulation between the carbon and Cu atoms. Interestingly, the adsorption of graphene on the (100) surface of Ni, Co and Cu is all physical and chemical mixed adsorption because there are the strong orbital coupling effect and the apparent charge accumulation between the carbon and metal atoms in some parts of these surfaces while there are almost no orbital coupling effect and charge accumulation between the carbon and metal atoms in the other parts. The results obtained by the present study are not only expected to explain the adsorption mechanism of graphene on the metal surfaces, but also to provide help for the research of graphene-based composites and carbon nanomaterials.

## Conflicts of interest

There are no conflicts to declare.

## Supplementary Material
